# A threat to the understanding of oneself: Intensive care patients' experiences of dependency

**DOI:** 10.3402/qhw.v8i0.20934

**Published:** 2013-06-28

**Authors:** Kristina Lykkegaard, Charlotte Delmar

**Affiliations:** Aalborg University Hospital, Aalborg, Denmark

**Keywords:** Intensive care, dependency, autonomy, shame, powerless, relation to the self

## Abstract

This study examines the meaning of dependency on care as experienced by intensive care patients. Literature on the subject is sparse, but research from nonintensive settings shows that dependency is often experienced negatively. The study is based on in-depth qualitative semistructured interviews with three former patients characterized as narratives. The analysis is inspired by a phenomenological hermeneutical method. The study has found that dependency is experienced as difficult and that the experience seems to be attached to the relationship to oneself. Patients feel powerless and experience shame, their understanding of self is threatened, and they fight for independence in the course after intensive care. The findings might be influenced by the study being conducted in a Western country setting, where independence is valued. They can be used as means of reflection on nursing practice and matters such as communication and patient participation.

The aim of this study is to explore the perceived meaning of being dependent on care for intensive care patients. Patients admitted to intensive care are a heterogeneous group admitted for very different reasons. But these patients share a high degree of dependency on others as their ability to perform self-care is reduced. Dependency is not typically verbalized by nurses, neither as part of their care actions nor in their collaboration with colleagues (Strandberg & Jansson, 2003). In other words, healthcare staffs do not know how dependency is experienced by patients, although dependency is often associated with negative feelings, powerlessness, frailty, and vulnerability (Henriksen & Vetlesen, 2000).

The subject has been investigated through qualitative in-depth interviews of lived experience with three former patients admitted to an intensive care unit at a Danish university hospital. The in-depth interviews have been characterized as narratives.

## Theoretical and empirical background

### Dependency in an ontological and cultural perspective

According to Henriksen and Vetlesen (2000), dependency determines what it means to be a human being. Dependency is linked to a resulting vulnerability, meaning that the human being who is referred to the care of others as a human being is not a self-sufficient self but is always in relationships to other human beings. In this referral, there is an inherent risk of not being seen and a risk that the caring needs of the individual are not met. Dependency thus contributes to making life frail (Henriksen & Vetlesen, 2000, pp. 31–33). Dependency on others is constant throughout life, and it is permanent, but it is often in connection with severe illness that we become conscious of this dependency on others. In this break that serious illness marks, we become aware of things that have functioned previously (Henriksen & Vetlesen, 2000, pp. 33–37). Dependency on others is often associated with negative feelings and powerlessness, and that is a value-laden concept. This can be understood from a cultural–historical perspective, as autonomy in the Western culture is considered an overall value. Autonomy means to be self-governing and self-determining. This interpretation of the concept of autonomy comes from the moral philosopher Kant (1724–1804) and still dominates today. To be self-sufficient financially and in relation to work as a cultural ideal can create a basis for the thought that we as human beings are independent. To be independent becomes an ideal demonstrating freedom that can be won by the human being. Dependency is considered something you can grow out of or detach yourself from. Thus, there is a tension between dependency as a basic human condition and the cultural ideals of individual freedom and independence (Henriksen & Vetlesen, 2000, pp. 37–46). In line with these thoughts, Løgstrup claims that there is an ontological dependency between human beings called interdependence. Human beings exist in a mutual dependency, and in one's meeting with another human being, power is inherent in all relations and interactions (Løgstrup, 1991).

### Experienced dependency in different health science contexts

There are several research studies on dependency on care in different contexts. Five studies explored how it is experienced to be dependent on help in one's own home. Ellefsen (2002) found that dependency limits the patients’ autonomy in connection with daily activities. Dependency on others is described as a burden associated with insecurity, low self-esteem, and lack of opportunity to negotiate the care. Monrad (2010) found that the help is a strain to some as it makes it difficult for the elderly person to maintain the life historical narrative of him or herself. The self is threatened, especially when the help takes over an activity that has defined the elderly person or when the help makes the limitations of the person clear. Dependency on others is also associated with powerlessness. In a PhD study, Lillestø (1998) found that people with functional limitations experience having to fight to maintain human dignity. Finally, studies focusing on people with Duchenne muscular dystrophy and people with spinal cord injury receiving respirator treatment in their home show that the correct help contributed to an experience of freedom (Dreyer, Steffensen, & Pedersen, 2010; Martinsen & Dreyer, 2012).

The experiences of dying patients have also been explored. Eriksson and Andershed (2008) have explored how it is experienced to be dependent on care in one's own home or at a hospice when death is imminent. They found that dependency is associated with experiences of bodily alienation, being a burden, and gratitude in relation to the professional caregiver. Dependency is described as a journey with difficult moments but also with periods of relief when the dying person receives the necessary help and experiences closeness with the caring staff.

Dependency has also been explored in the hospital sector. In a study of medical and surgical patients, Strandberg, Norberg, and Jansson (2003) found that dependency on care involves a struggle to receive the help needed, and the context, degree, and type of dependency may influence how dependency is experienced. Finally, Lomborg, Bjørn, Dahl, and Kirkevold (2005) conducted a study on how patients with chronic obstructive pulmonary disease experienced receiving help for bodily care. They found that dependency on others was experienced as a threat to the patients’ integrity and that negative aspects of receiving help dominate. They preferred when nurses took the initiative.

### Dependency in the intensive care patient

The review showed that literature is sparse on the dependency of intensive care patients and that the majority of studies focus on how dependency can be estimated to assess the optimal nurse–patient ratio, the effect of different classification systems for this assessment, or an estimation of the workload of nurses (Adomat & Hewison, 2004; Adomat & Hicks, 2003; Donoghue, Decker, Mitten-Lewis, & Blay, 2001; Large, Nattrass, & Simpson 1991; Wardle, 1997). These articles thus investigate dependency from an organizational perspective.

Three articles discuss different aspects of dependency on care from a patient perspective. In an older article, Griffin (1982) discussed how the self of critically ill patients is threatened when they are forced to be dependent on others. Perception of self is described as an important part of perceiving oneself as whole, and it is important to the perception of security and self-esteem. Griffin described how nursing and doctor activities force patients to realize how seriously ill they are, and this changes their perception of the self. There is an experience of loss in this, and the perception of wholeness is disturbed. Intensive care patients are forced into this dependency, which is passively related to losing control of daily activities and having their privacy invaded by others. Forced dependency and loss of control are described as significant stressors in the intensive care patient; some patients describe the feeling of lack of control and helplessness as more threatening than the threat of death. The article is rather old, and it is uncertain whether it is based on actual research as it has not been possible to procure the sources on which it is based.

Two newer studies from the intensive care setting describe dependency, but none of the studies have particularly focused on dependency. McKinley, Nagy, Stein-Parbury, Bramwell, and Hudson (2002) studied the experience of being a critically ill patient at an intensive care unit. The study was based on focus group interviews with 14 former patients 3–6 months after discharge. The study found that vulnerability is the concept that best characterizes the intensive care patient. Vulnerability is related to the patient's extreme dependency during critical illness and lack of ability to meet one's own needs. The vulnerability is aggravated when, for example, these needs are not met by the staff. When needs are met and the care is individualized, patients experience a sense of security despite their vulnerability. It seems to be important to the patients that nurses can anticipate their needs. Almerud, Alapack, Fridlund, and Ekebergh (2007) corroborated these findings where they explored how it is perceived to be critically ill or injured in a high-technology intensive care unit. The study was based on interviews with nine patients during their admission or shortly after discharge. Dependency is one of the elements that defined the experience of being an intensive care patient. Patients felt monitored but invisible; the incomprehensible environment and technology limit the patients, lead to passive behavior, and compromise the patients’ integrity. Control, influence, and freedom disappear, and patients leave themselves to others; this leads to contradictory feelings of security and vulnerability. Dependency on others and technology are described as a form of suffering where the patients have no other choice but to surrender to machines and routines. The study showed that patients try to be good patients by adapting to the expectations of the system and that technology comes between the patient and the nurse.

The literature review on intensive care patients and dependency has shown that existing studies primarily focus on dependency from an organizational perspective. A few studies have described how dependency is part of the experience of being an intensive care patient. Dependency is in these studies described as being associated with major vulnerability and suffering. However, there are no empirical studies focusing on experienced dependency. As patients are increasingly awake during their stay at the intensive care unit as a consequence of altered sedation practices, it can be assumed that patients are more conscious of dependency on others compared to previously.

The reviewed literature from nonintensive settings show that dependency leads to many different experiences for patients but that dependency often is experienced negatively. This could indicate that the context plays an important role for how dependency is experienced. Dependency on care will presumably not be experienced in the same way at an intensive care unit compared to receiving help in the home or in another hospital context. Care seems to be more easily accessible in an intensive care unit as it is not planned in the same way as in the primary care field, and the staffing is higher compared to in ordinary bed wards. Patients on respirator treatment are special because they can have difficulties communicating their own needs. The studies reviewed from nonintensive settings show that dependency is associated with insecurity, powerlessness, lack of control, limited autonomy, bodily alienation, and threatening of integrity and self as a consequence of one's dependency on others. Dependency is described as being linked to suffering, violation, and fighting where the patients are subjected to the treatment of others. Research also shows that patients use different strategies to cope with dependency. There are moments of relief, too. It thus seems relevant to focus on the meaning that intensive care patients attach to being dependent on care.

## Study design and methods

The study is based on three in-depth interviews following the participants’ narratives. With a phenomenological hermeneutic approach, data were collected through semistructured questions (Brinkmann & Tanggaard, 2010; Kvale & Brinkmann, 2009; Tanggaard & Brinkmann, 2010), and the analytical method is inspired by Lindseth and Norberg (2004). Their method has been used in several studies and is applied in healthcare research and human studies when it is important to obtain knowledge of the meaning of lived experience. The main inspiration for developing their method is Ricoeur's phenomenological hermeneutical interpretation theory of moving through three methodological steps in the analysis and interpretation of the text, which is carried out on three levels: a naive reading, a structural analysis, and a comprehensive understanding (interpreted whole). The intention with the method is to improve understanding of a phenomenon. It can be used when studying phenomena such as dependency (Dreyer & Pedersen, 2009). Through the patients’ lived experiences and the resulting narratives, the researcher tries to obtain a possible meaning of being dependent on care.

### Selection of participants

Participants were recruited from a high-technology intensive care unit of a larger Danish university hospital receiving medical and surgical patients.

A purposeful selection of the three participants (Polit & Beck, 2010, pp. 319–324) was made based on the following arguments: it is known that many intensive care patients have recall problems. Prolonged admission and respirator treatment have been shown to influence the ability to recall the stay and the respirator treatment, including the experiences attached to nursing care (Bergbom-Engberg, Hallenberg, Wickstrom, & Haljamae, 1988; Capuzzo et al., 2001). To minimize some of the recall problems, criteria are saying that patients must have been discharged from the intensive care unit within the last 6–12 months and have received respirator treatment for 1 week or longer. Because of the recall problems, it is very difficult to recruit participants.

Only adults older than 18 years interested in telling their experiences were selected. We assume that there will be other experiences of dependency among children and adolescents. Moreover, only rarely are patients younger than 18 years admitted to the intensive care unit where participants from this study were selected. As patients in all age groups are admitted to an intensive care unit, it was in addition to methodological considerations deemed relevant with an age span. Only persons who could speak and understand Danish and have a Danish cultural background participated. This seems relevant as dependency is considered a value in Western society (Henriksen & Vetlesen, 2000). Finally, the participants were interviewed after discharge considering ethical issues concerning their well-being, as research has shown that intensive care patients are strongly affected emotionally by the intensive care experience during the course after discharge to the bed ward (McKinney & Deeny, 2002).

Patients with signs of posttraumatic stress or patients who had been mentally unstable during the admission to the intensive care unit did not participate in the purposeful selection. The head nurse knew of two potential participants with signs of posttraumatic stress and one patient who was admitted due to attempted suicide. These patients were not asked to participate.

Through the head nurse at the intensive care unit, contact was established to the participating patients. The nurse was in telephone contact with former patients who complied with the purposeful criteria. The participants were briefly informed about the study by the head nurse, and if they were interested in more information, a letter was sent providing it. Subsequently, participants were contacted by telephone for confirmation of participation and to make an appointment for the time and place of the interview. The written material suggested that the interview take place in the participant's home, but each participant made his or her own decision.

It was difficult to find participants for the study. Eight persons were contacted by the head nurse at first; three agreed to participate. The remaining five were all males; two had no recollection of the stay at the intensive care unit, one declined due to lack of time, one never replied, and one was in such a poor state that participation was not possible according to his spouse. Several of the patients who, according to the department nurse, were candidates for the study had died after discharge from the intensive care unit. Thus, the criteria for discharge were changed to between 3 and 12 months. The three participants were:

- Participant 1: 27-year-old female admitted to the intensive care unit due to surgical complications. The admission lasted 3 weeks. The woman subsequently stayed for a prolonged period of time at a surgical bed ward and has needed help from her family after discharge from the hospital. She was interviewed 1 year after admission to the intensive care unit, and at this time she had resumed her job.
- Participant 2: 78-year-old woman admitted to the intensive care unit due to a complicated postoperative course. Moreover, the woman had a known medical problem, which had made the treatment difficult. She was admitted for 3 months and, subsequently, she participated in prolonged rehabilitation; the total length of hospital admission was approximately 6 months. She was interviewed 3 months after discharge from the intensive care unit, when she had just come home. She still needs help in the home; the help is provided by family and the professional system. During the stay at the intensive care unit, the woman had been transferred to a smaller hospital with a smaller intensive care unit, and her recollections are primarily from the latter unit. The woman is a pensioner.
- Participant 3: 56-year-old male admitted to the intensive care unit due to a medical problem. The stay lasted 3 weeks, and subsequently he was admitted to a medical ward. He was interviewed approximately 3 months after discharge from the intensive care unit. At this time, he is temporarily staying at a nursing home. He has been an early retirement pensioner for 15 years.

The participants had been used to managing on their own before the admission to hospital. During the stay at the intensive care unit, they have needed comprehensive help “for everything.” In time things have developed, especially for participant 2, who says that she was able to feed herself during the last course of the admission. The participants had difficulties recalling the time at the intensive care unit and to make coherent narratives; especially participant 3 had difficulties distinguishing dreams from reality and episodes from the bed ward and the intensive care unit. It has been possible to exclude parts of the interview due to knowledge of the special characteristics of the intensive care unit. It has not had any immediate consequence for the number of recollections when the participants were interviewed; this is in line with research in the area (Löf, Berggren, & Ahlström, 2006).

### Materials and analysis

The interview guide contained the following themes: dependency in relation to understanding of self, dependency in relation to time, bodily experiences linked to dependency, and experiences in relation to the nurse. Themes and questions were based on the literature review. An analysis of the concept of “care dependency” (Boggatz, Dijkstra, Lohrmann, & Dassen, 2007) furthermore inspired the questions as they recommend that the empirical studies should include questions about the patients’ functional limitations, which support the patients needed, and if the patients received this support. The guide has functioned only as an indicator. Above all, it is the patients’ narratives and experiences that have been followed.

The interviews were recorded on an audio file, transcribed verbatim, and decontextualized to a text that could be analyzed (Kvale & Brinkman, 2009, pp. 199–209). Transcriptions have been made after each interview to provide a clear recollection of the interview; to increase the reliability, parts of the interviews have been heard many times. The transcription process has been long and has demanded carefulness.

The findings appear through an analysis and interpretation of the text, which is carried out on three levels: a naive reading, a structural analysis, and an interpreted whole that can increase, change, or broaden the understanding. This means that it is a continuous process from what is said to what is talked about. In the naive reading, the text is read several times to grasp its meaning as a whole (Lindseth & Norberg, 2004). This demands a phenomenological approach where the researcher is open to what the text says and becomes influenced and moved by it. In this study, the naive reading and the immediate impressions in relation to “what is said” were, for example, “I became a whole different person,” “then you have to eat humble pie and ask for help, right,” and “it was really overstepping boundaries.” The naive reading is characterized by guessing, which is inherent in the text, and it will subsequently be validated or rejected in the structural analysis.

The structural analysis searches for themes, threads of meaning penetrating the whole text or parts of it. These meaning units are read and reflected upon in relation to the naive reading. Subsequently, they are condensed, which means that the essence of each meaning unit is expressed as precisely as possible using everyday language. Reflections are made on the similarities and differences of the meaning units, and a further condensation is made into themes and possibly subthemes. Finally, reflection is made on the themes in relation to the naive reading. If this cannot be validated by the themes, a new naive reading is made and a subsequent structural analysis. This process continues until the naive reading can be validated. A text can include several meanings, and more structural analyses might be necessary to unfold these (Lindseth & Norberg, 2004). It is an iterative process where the interviews are read several times and in different ways to achieve an understanding of what is said and explain a possible meaning. During this iterative process, different themes have been rejected as the repeated readings have shown that something else was in play in the texts. The preunderstanding has been challenged in discourse with each other and with other healthcare providers. The final themes are the researchers’ interpretation of what the quote refers to and the quotations providing the justification hereof. The quotations have been made reader friendly, which means that repetitions, which are part of the spoken language, have been removed when they neither directly nor indirectly had any importance for what was said.

Moreover, it has an ethical aim as verbatim transcriptions can seem stigmatizing if the reader is not conscious of the difference between spoken and written language (Kvale & Brinkmann, 2009, pp. 206–210). Parentheses in quotations explain what the participants talk about when the quotations are taken out of a longer, coherent talk about a subject.

The themes are reflected on with literature texts in the discussion section with the goal of interpreting the text as a whole and arriving at a comprehensive understanding of being a dependent intensive care patient. Table I is an example of a structural analysis.

**Table I:** An example of structural analysis.

Meaning unit	Condensation	Subtheme	Theme
It hit me so much being that dependent on others because I have never been that before. I have always lived alone and managed by myself, taking care of myself and others. (participant 1)	Grieving the loss of the known self.	To be dependent on care influences understanding of self.	With dependency and critical illness, the relation to the self is changed.
It was myself I was angry with because I thought I was a failure that something, well, I couldn't do it, which in daily life is a small detail … I felt like a little baby when I needed help to eat, get washed and get rid of my excretions. (participant 3)	Ashamed of what you can no longer do. Sign of weakness. Your honor is at stake.	It is associated with shame to receive help for care.	With dependency and critical illness, the relation to the self is changed.
All the strength I had disappeared and I lost an incredible amount of muscle mass. My left eye was very watery and I could not lift my arm to dry away the tears. The powerlessness was that I needed someone to dry away a tear. It was just a small thing that I under normal circumstances would never have given a thought … but I had to do that … at least at this time I had to deal with everything and let others take care of me. I hope I will never experience this again. (participant 1)	Forced to receive help. Not having the situation and the body under control.	You feel powerless during critical illness.	With dependency and critical illness, the relation to the self is changed.

### Ethical considerations

An interview is a moral investigation, and ethical and moral issues should thus be inherent in all phases of the interview study (Kvale & Brinkmann, 2009, Chapter 4). Interview knowledge is an asymmetrical power relation as the interview is defined and instrumentalized by the researcher. It is important that the researcher reflects on the significance of this power in relation to the knowledge produced (Kvale & Brinkmann, 2009, pp. 50–52). During the interviews, it has been attempted to follow the participants’ narratives and let them finish talking before asking any elaborating questions, showing respect for the participants’ integrity. Also during the analysis, the interview texts have been carefully considered if interpretations are rooted in the participants’ narratives.

The interviews lasted between 45 and 60 min. Participants have spoken openly about their experiences, and it was the impression that the relation to the participants was good and they experienced the situation as safe. Everybody during the subsequent briefing expressed that participation in the interview was positive.

Before the interview was finished, the participants were asked if they had further experiences they wanted to share. This was the case for two of the participants. Two interviews were conducted in the participants’ homes; one interview, upon request from the participant, was conducted in an undisturbed meeting room at the hospital. During one of the interviews, the spouse of the participant was present in accordance with the participant's wish and contributed when the memory of the participant was weak concerning admission dates and illness history. Moreover, the spouse has helped the participant recall experiences that the participant at other occasions had reported being important. At the remaining interviews, only the interviewer and the participant have been present; in one interview, though, there were several disturbances during the interview as the participant was at a nursing home. The tape has in these situations, except for one, been stopped. In this one case, the participant wanted to continue the interview despite the brief presence of the nurse. The participant did not seem to be influenced by this. If the participants needed to discuss the interview further, they had the interviewer's phone number.

The national ethics committee has decided that an ethical approval was not necessary, as the study did not include biomedical aspects. The study complies with Danish legislation and is approved by the Danish Data Protection Agency. All information on participants has been dealt with confidentially, and sound files and interview transcription files were given an ID number and stored separate from sensitive personal data. Prior to each interview, a written and oral informed consent was obtained, and it was made clear that participation was voluntary and that it was possible to leave the study at any time without further explanation. The participants were also informed that they would be anonymous and that information would be handled confidentially. All participants signed a consent form. Moreover, the study was made in accordance with the ethical guidelines for nursing research in the Nordic countries (Sykepleiernes Samarbeid i Norden [SNN], 2003).

## Findings—with dependency and critical illness, the relation to the self is changed

In the naive reading, patients describe depending on help for care when admitted to an intensive care unit as unusual, embarrassing, crossing boundaries, and associated with shyness and the feeling of being a burden; shame, dignity, and violations seem to be pivotal. The descriptions of dependency go beyond the stay at the intensive care unit and connect the past, present, and future. In the description of how dependency is experienced, there is thus a reference to how you were previously, a reference to the present, and one to what the future will bring. The patients’ self-understanding seems to be in play with dependency, and there is a fight to be free of this and regain independence again.

Attached to the patient's illness is also a bodily change that has caused the need for help and is connected to the experience of powerlessness. Powerlessness can also be caused by the difficulty or even inability to communicate needs due to respirator treatment. The bodily change and the powerlessness can cause death to be perceived as an easier alternative, or can lead to putting up with needs not being met. In addition, the bodily change leads to a different consciousness of the body from usual. The experience of being dependent changes over time, but it is also perceived differently by participants, maybe because of their different courses of admission and varying age.

In the structural analysis, the theme “With dependency and critical illness, the relation to the self is changed” is expressed in the following subthemes: “To be dependent on care influences self- understanding,” “It is associated with shame to receive help for care,” and “You feel powerlessness during critical illness.”

### To be dependent on care influences understanding of self

*For the participating patients*, the narratives of the perceived dependency have led to stories about who they are. To be dependent on care can thus seem too important to the understanding of the self. There are reports of how dependency and the bodily change are perceived as difficult and unusual compared to the way they have acted previously or the roles they have had. Dependency thus changes you. Participant 2 expressed:

… you know it is a chapter of its own (to be dependent on care). I am stubborn and I have always wanted to manage myself. And then, just like that you are dependent on others in a very annoying way … once they called me the lightning because I was fast and suddenly the lightning is out. Oh, by God it is annoying … I was a whole different type so damn it, it's difficult.

From these expressions, it is obvious that dependency is very difficult, and by the expression “damn it, it's difficult” you get the feeling of grieving the loss of the known self. Participant 1 says something similar and refers, among other things, to the role she had previously as the helper:

It hit me so much being that dependent on others because I have never been that before. I have always lived alone and managed by myself, taking care of myself and others.

With the expression “that dependent,” you could presume that the strong significance that dependency has for the participants is attached to the seriousness of their disease and the fact that they had extensive needs for care during their admission to the intensive care unit. Participant 3 talks about dependency in relation to who he was previously:

I was annoyed and angry with myself because I couldn't do anything. I have been used to managing on my own since I was a kid, right? I could do everything and all this stuff.

Dependency is in this way attached to difficult feelings, and it seems to interfere with the way you understand yourself.

The experience of dependency also goes beyond the interaction with the care staff. As time went by, participants 2 and 3 have learned to accept help. Participant 2, for instance, says that she has learned to receive help, but it has been a dramatic change as she has been used to subject herself to a lot. Participant 3 also says,

The most difficult hurdle to overcome, I believe actually it's like that in many ways, is self-awareness – that you have to accept this is what the situation is like right now – and get the best out of it.

To participants 2 and 3, it has in time become easier to be dependent. Participant 1 describes the opposite. To her, it was easier to receive help in the beginning when she was very ill. The reason for this difference is unknown. It might be of importance that participants 2 and 3 have been dependent on help during a longer period, and they have had to acknowledge the need for help. It might also play a role that they both had difficulties recalling the time at the intensive care unit. The experience of dependency seems, however, to be changeable. It should also be noted that all participants, despite acceptance, seemed to struggle to regain power and independence and in this way return to their usual life. It was a fight that goes beyond the stay at the intensive care unit. Participant 3 says, “I grow stronger and stronger every day, I think myself, but I also fight and do something for it,” and participant 1 says, “Maybe it took me 25 minutes to tie a shoe lace, but I would do it myself. It was also a counter reaction to me having been so dependent on others.” Thus, participants want to free themselves from the dependency.

### It is associated with shame to receive help for care

For the participating patients, it has been difficult to receive help, and especially body care has been difficult. It seems to be associated with shame to receive help, as participants talk about it as being degrading, embarrassing, and a sign of weakness. Shame is linked to being undressed but also to being ashamed of what you can no longer do. In the literature, there are several examples indicating that receiving help can mean that you are at odds with who you think you should be. Participant 2 mentions that personal pride makes it difficult to receive help. This statement could indicate that your honor or value is at stake when you receive help. Participant 3 also talks about being ashamed of what you no longer can do:

It was myself I was angry with because I thought I was a failure that something, well, I couldn't do it, which in daily life is a small detail … I felt like a little baby when I needed help to eat, get washed and get rid of my excretions.

And he refers to what he cannot do and says that he “should” be able to do it. A major need for help during a stay at the intensive care unit seems to impact the strong feelings and the experience of lacking value. Participant 1 similarly links dependency with a distinction between child and adult. Participant 3 twice during the interview, although in different contexts, refers to what he himself calls a moral: “… that I just let others do the job is not really me,” and “… in my moral right it is like I help as much as I can, right?”. The statements indicate that the participant sees a connection between being independent and participating and moral, while being dependent and not able to care for himself could mean that you are at odds with you own moral standards and with who you think you are supposed to be. Similar ideas can be found with participant 1, who sees dependency as a sign of weakness and lack of independence.

Finally, the shame of receiving help does not seem to be associated with staff's way of reacting but exists in spite of this. The participants praise, for instance, the nurses for being kind, and participant 1 says, “They can hop and dance and jump around like crazy but it doesn't change the fact that they still have to clean my butt.”

### You feel powerless during critical illness

When you are critically ill, there is a feeling of powerlessness. The powerlessness is due to the bodily changes and the lack of ability to communicate. A part of the powerlessness seems to be a resignation in which you put up with or defer any needs as you are helpless and have no possibility to act. The feeling of powerlessness is clearly expressed by participants 1 and 3 but is also found in participant 2. It is unknown why there is this difference, but a possible explanation could be that participant 2 had a longer intensive course with ventilator weaning where she to a larger extent than the other participants had an influence on her own care and treatment. Participant 1 expresses the feeling of powerlessness in the following quotation:

All the strength I had disappeared and I lost an incredible amount of muscle mass. My left eye was very watery and I could not lift my arm to dry away the tears. The powerlessness was that I needed someone to dry away a tear. It was just a small thing that I under normal circumstances would never have given a thought … but I had to do that … at least at this time I had to deal with everything and let others take care of me. I hope I will never experience this again.

The powerlessness was linked to the changes in the body, and it was experienced very intensely and meant that she was forced to receive help. The meaning unit also indicates that the body is made conscious in a different way from normally, as activities that normally are matters of course suddenly need consideration. The body can seem strange, which is underlined by the participant saying that she has found it difficult to feel her body and make it react as she is used to. Participant 3 says something similar:

… and then you just lie there and you just can't (get up) and you feel completely helpless, right? You fight and you fight and you fight just to lift your arms or move your legs and then at last (exhaling) then you have to come clean and ask for help right … it is so frustrating.

In this meaning unit, the powerlessness seems to be attached to the changes of the body and leads to some difficult feelings. There seems to be a humiliation in the expression “to come clean,” and you could get the impression that the need for help is difficult to accept. Participant 3 also thought the body was strange as he told he could feel he was “far out” and when he in his mind had strength as usual but the body was all “wobbly.”

Finally, the powerlessness is expressed in connection with communication problems during respirator treatment. Communication problems were some of the worst parts of being at the intensive care unit. Participant 3 returns to this several times during the interview when he says:

You couldn't tell what you wanted if you wanted something else than the big heavy duvet to cover you, right … you can reach this kind of desperation right – understand what I'm saying.

He finishes by saying that in the end you give up or postpone your needs. He experiences being without influence. Resignation seems to be linked to powerlessness as the participant gives up, which was also expressed in the interview with participant 1. She tells repeatedly about uncomfortable care where she puts up with it because does not think she has other options. The powerlessness in these quotations seems to be associated with the severity of the disease. She says:

… you are really far out, now you have to find yourself and trust that they know what is best and if that means you have to lie there with your nacked butt while they treat the socalled bed ulcer then you have to (stay lying down). And then it was kind of no longer there it was not in my head any longer. Suck it up. I wouldn't have done that “today” and “later” … I could not remove myself from a very uncomfortable situation. I could just keep lying there … I put up with everything because I felt it was the only choice I had.

It is clear from the statements that the experiences have been intense and have crossed some boundaries for this participant and that she reacts differently from usually. Finally, the bodily change and not being able to care for yourself have meant that the spirit of life at some time was lost during the stay at the intensive care unit and that death could be an easier alternative; this was very concretely expressed by participant 2:

I was mad at it (the body). And it didn't help (laughing). I have to be honest, there was a time during my stay at the intensive unit where I thought that if I needed an injection of some kind then couldn't they just give me a proper dose and then I was gone (crying).

She does not talk directly about the feeling of powerlessness, but it seems to be inherent in the resignation in the meaning unit.

## Discussion

With the goal of arriving at a comprehensive understanding of being a dependent intensive care patient, the three subthemes are summarized one by one and reflected on in relation to the aim of the study and by comparing the findings with literature.

To be dependent on care seemed to interfere with the patients’ self-understanding and dependency, and the bodily changes were perceived as difficult and unusual compared to the way they acted previously and the roles they had. The patients are changed by dependency, and it seems there is a loss of the known self connected to this. Dependency was a radical change that patients found hard to accept, and they struggle to regain strength and independence afterward.

Identity is, according to Horsdal (1999), a narrative construction (i.e., a story). It is the story of who you are in relation to the life you have lived and the wishes you have for the future. Identity is about creating coherence and making sense of what is separated by time. Identity is individual but also cultural as the human being enters into social and historical contexts. When something fatal happens that collides with your expectations, the vulnerability of the self appears and meaninglessness can occur. Establishing a revised story makes it possible to move on (Horsdal, 1999, pp. 69–80). When patients talk about who they were, and their thoughts about the future at the same time as they speak about dependency, it can be understood as if identity with dependency and critical illness has been threatened. The story can be understood as an attempt to create meaning. Two patients who learn how to accept this might indicate that the personal story has been revised and that experiences are integrated in the identity. Also, Monrad (2010) found that receiving help can challenge the self. The strain felt by participants is more linked to the life story than to specific situations, and the strains go beyond the interaction with the caregiver. The relation between health changes and the self of the individual was important to how receiving help was perceived; thus, it is different from what is perceived as strenuous.

Considering Monrad (2010) and Horsdal (1999), it can be summed up that to some it can be perceived as threatening to the known self and the identity to be dependent on help when the help interferes with the way you are used to understanding the self. It is therefore possible that patients who live in a different way from that of the participants interviewed, who are used to receiving help, or who do not attach importance to independence to the same extent perceive dependency in a different way.

The findings of the study also seem to be in contrast to the understanding of dependency as a basic human condition as described by Henriksen and Vetlesen (2000). This is further emphasized by participants fighting for independence. A finding confirmed by Strandberg et al. (2003) described how medical and surgical patients try to regain lost abilities through hard training and how it is experienced as being of major importance to manage personal care. Ågard, Egerod, Tønnesen, and Lomborg (2012) describe, in a study of patients’ worries and coping strategies during the first 12 months after discharge from an intensive care unit, how patients fight for independence and focus on regaining physical strength, functional capacity, and domestic roles. Patients do not worry about traumatic events or psychological consequences. Dependency thus seems to be a condition many patients wish to be free of.

The analysis also points to the feeling of shame. It is considered degrading to receive help for personal care and a sign of weakness that you are no longer able to take care of yourself. In the narratives, the shame seems to go beyond the interaction with the staff and be considerably connected to the relation to self and to dependency colliding with who you think you should be.

The nursing theorist Martinsen writes, in regard to shame, that it is attached to person and identity, and when being ashamed you are judgmental to yourself. You do not live up to standards and norms attached to your identity, and the dignity is destroyed (Martinsen, 2012, pp. 76–79). She writes on the basis of Vetlesen about a new shame created by society where the responsibility of the individual for their own situation is stressed and where, for example, independency, coping, and control are desirable resources. Dependency is associated with taboo and shame and with the new shame; the life force can be destroyed if you do not meet the demand of self-sufficiency (Martinsen, 2012, pp. 90–100). That patients’ experience of shame may be linked to what Martinsen calls the new shame and the fact that they, during the stay at the intensive care unit, have had a comprehensive need for help. It might also be linked to the new shame that patients can lose their zest for life during their stay at the intensive care unit when the body does not function as usual. The cultural demand for self-sufficiency cannot be met for the participants, and this is perceived as losing value as a human being. Martinsen writes that it is a challenge for health professionals to consider the reality of the cultures we are a part of in order to accept the mutual dependency we have on each other as human beings and accept that the human being is valued (Martinsen, 2012, pp. 98–100). This is a challenge that is particularly relevant, as a report from Dansk Sundhedsinstitut (2010) on caring tasks in the Danish healthcare system shows that future hospitalized patients will be more ill and less able to help themselves. There are no other studies describing perceptions of shame in the intensive care setting. This could be because dependency on care has not been studied in this group of patients previously, and this finding must be validated in future research. It should be mentioned that Schou and Egerod (2008), in a study on the perceptions of intensive care patients when ventilator weaning, found that patients perceived it as embarrassing to not be able to communicate as usual. This could indicate that shame is perceived by the intensive care patient when the possibilities to act during critical illness are changed. This finding is supported by a study on terminal patients’ dependency on care where shame is expressed as a consequence of the body not functioning as previously, which can be linked to the dying feeling left outside the community (Eriksson & Andershed, 2008).

The intensive care patient also experiences bodily changes and a feeling of powerlessness, for example, because it can be difficult to communicate one's own needs. The powerlessness can be experienced as very dramatic, and it seems to be associated with resignation: putting up with, for example, uncomfortable care actions or giving up on having needs met after experiencing being helpless and without the possibility to act. The bodily changes can also mean losing zest for life during one's stay in the intensive care unit. According to the Danish philosopher K. E. Løgstrup, human conditions are always relations of power, meaning that it is inevitable that human beings to some extent are referred to each other (Løgstrup, 1982, 1991). Human beings exist in a mutual dependency, and in the meeting with another human being you will thus always have a part of the other person's life in your power, and it is thus an ethical demand to take care of this. According to Løgstrup, any human being is also independent and responsible, and the responsibility for the other is not about taking away independence from the other (Løgstrup, 1991, pp. 33–39). The contrast to independency is authority, and it gets its life from authority. Thus, it is in one's independence that you can place authority on others, and authority is therefore to be understood as something positive. If the person, on the other hand, gives up independence, the authority becomes authoritarian, which according to Løgstrup is a deprived authority as the initiative is moved and the relation to the other becomes blind obedience (Løgstrup, 1991, pp. 65–70). The patients’ perception of powerlessness can be interpreted on the basis of these thoughts by Løgstrup. The feeling of powerlessness can be understood as an expression of what the independent person is forced to give up as critical illness takes the initiative and ability to communicate becomes difficult. In this way, the other is not voluntarily assigned authority and the relation to the caring staff could be perceived as authoritarian. This could also explain what the participants put up with and how they deferred their needs, and it could be an expression of what Løgstrup calls blind obedience. Powerlessness seems to create an imbalance in the power relation where all power is given to the caregiver.

Delmar (2011) calls this kind of powerlessness a relational ethical life phenomenon but supplements this understanding with individual existential life phenomena. To the individual patient, powerlessness can be characterized as life constrained in connection with illness, where life phenomena become more marked. When patients lose their zest for life, the powerlessness can be expressed as a life-constraining phenomenon. According to Delmar, it is important that the powerlessness does not take control and that the ill person is helped to manage life. This could be by creating room for life-conducive phenomena such as hope and spirits.

Other research also describes perceptions of powerlessness. A new study, Karlsson, Bergbom and Forsberg (2012) describes how awake patients receiving respirator treatment perceive it as being in a negative relation of dependency where you are conscious of your own helplessness and do not have the possibility to act. That patients do not act is linked to the lack of ability to control time, position, and space as a consequence of not functioning as usual. It is also described how lack of information increases one's helplessness, and the experience of powerlessness arises as a consequence of communication difficulties. Samuelson (2011) has also described perceptions of insecurity, powerlessness, and helplessness in intensive care patients. This is associated with lack of awareness of what has happened, loss of information, and lack of ability to manage on your own.

The above-mentioned studies can, in line with the findings of this study, confirm that powerlessness is a fundamental feeling that can occur in intensive care patients. It seems to be important that staff in intensive care units improve communication with the patients and create room for life-conducive phenomena as these factors seem to influence the perception of powerlessness.

## Conclusion

The purpose of this study is to explore the perceived meaning of being dependent on care for intensive care patients. The study is relevant on the basis of the literature review that has shown that this aspect of the intensive care experience is only scarcely studied.

It can be concluded that patients perceive it as difficult to be dependent on care. The feeling of dependency is related to the individual's relation to the self, which is changed through dependency and critical illness. It is an interesting aspect that the perception of dependency goes beyond the interaction with the caring staff and that this is of major importance for the participants’ self-understanding, which seems to be threatened. It is the loss of the known self and the experience of shame as a result of not being able to take care of yourself. It is a shame that could be caused by a society valuing independence and self-sufficiency. The patients perceive powerlessness as a consequence of the bodily change—a powerlessness that can be explained by being forced to give up independence but that can also be understood as an existential life phenomenon that becomes evident as a result of changed conditions of life.

The study seems to reveal that there is an existing struggle to regain power and independence, particularly afterward, which indicates that dependency is something you need to free yourself from. The participants do not seem to view dependency as a basic human condition.

## References

[CIT0001] Adomat R, Hewison A (2004). Assessing patient category/dependence systems for determining the nurse/patient ratio in ICU and HDU: A review of approaches. Journal of Nursing Management.

[CIT0002] Adomat R, Hicks C (2003). Measuring nursing workload in intensive care: An observational study using closed circuit video cameras. Journal of Advanced Nursing.

[CIT0003] Ågard A. S, Egerod I, Tønnesen E, Lomborg K (2012). Struggling for independence: A grounded theory study on convalescence of ICU survivors 12 months post ICU discharge. Intensive Critical Care Nursing.

[CIT0004] Almerud S, Alapack R. J, Fridlund B, Ekebergh M (2007). Of vigilance and invisibility—being a patient in technologically intense environments. Nursing in Critical Care.

[CIT0005] Bergbom-Engberg I, Hallenberg B, Wickstrom I, Haljamae H (1988). A retrospective study of patients’ recall of respirator treatment (1): Study design and basic findings. Intensive Care Nursing.

[CIT0006] Boggatz T, Dijkstra A, Lohrmann C, Dassen T (2007). The meaning of care dependency as shared by care givers and care recipients: A concept analysis. Journal of Advanced Nursing.

[CIT0007] Brinkmann S, Tanggaard L, Tanggaard L, Brinkmann S (2010). Introduktion [Introduction]. Kvalitative metoder: en grundbog *[Qualitative methods: A basic book]*.

[CIT0008] Capuzzo M, Pinamonti A, Cingolani E, Grassi L, Bianconi M, Contu P (2001). Analgesia, sedation, and memory of intensive care. Journal of Critical Care.

[CIT0009] Dansk Sundhedsinstitut (2010). Fremtidens plejeopgaver i sygehusvæsenet. Notat: Sidsel Vinge *[Caring tasks in the Hospital System of the future. Document: Sidsel Vinge]*.

[CIT0010] Delmar C (2011). Beyond the drive to satisfy needs—in the context of health care. Medicine, Health Care & Philosophy.

[CIT0011] Donoghue J, Decker V, Mitten-Lewis S, Blay N (2001). Critical care dependency tool: Monitoring the changes. Australian Critical Care.

[CIT0012] Dreyer P. S, Pedersen B. D (2009). Distanciation in Ricoeur's theory of interpretation: Narrations in a study of life experiences of living with chronic illness and home mechanical ventilation. Nursing Inquiry.

[CIT0013] Dreyer P. S, Steffensen B. F, Pedersen B. D (2010). Living with severe physical impairment, Duchenne's muscular dystrophy and home mechanical ventilation. International Journal of Qualitative Studies on Health and Well-Being.

[CIT0014] Ellefsen B (2002). Dependency as disadvantage—patients’ experiences. Scandinavian Journal of Caring Sciences.

[CIT0015] Eriksson M, Andershed B (2008). Care dependence: A struggle toward moments of respite. Clinical Nursing Research.

[CIT0016] Griffin J (1982). Forced dependency in the critically ill. Dimensions of Critical Care Nursing.

[CIT0017] Henriksen J, Vetlesen A. J (2000). Omsorgens etik: grundlag, værdier og etiske teorier i arbejdet med mennesker *[The ethic of caring: Ontology, values and ethical theories in working with human beeings]*.

[CIT0018] Horsdal M (1999). Livets fortællinger: en bog om livshistorier og identitet *[The stories of life: A book focusing on life stories and identity]*.

[CIT0019] Karlsson V, Bergbom I, Forsberg A (2012). The lived experiences of adult intensive care patients who were conscious during mechanical ventilation: A phenomenological-hermeneutic study. Intensive Critical Care Nursing.

[CIT0020] Kvale S, Brinkmann S (2009). Interview: introduktion til et håndværk *[Interview: An introduction to a handcraft]*.

[CIT0021] Large W. P, Nattrass M, Simpson M (1991). Working towards dependency scoring in critical care. Intensive Care Nursing.

[CIT0022] Lillestø B (1998). Når omsorgen oppleves krenkende: en studie av hvordan mennesker med fysiske funksjonshemninger opplever sitt forhold til helsetjenesten *[When caring becomes violating: A study of how human beeings with physical limitations experience the health care system]*.

[CIT0023] Lindseth A, Norberg A (2004). A phenomenological hermeneutical method for researching lived experience. Scandinavian Journal of Caring Sciences.

[CIT0024] Löf L, Berggren L, Ahlström G (2006). Severely ill ICU patients recall of factual events and unreal experiences of hospital admission and ICU stay—3 and 12 months after discharge. Intensive and Critical Care Nursing.

[CIT0025] Løgstrup K. E (1982). System og symbol *[System and symbol]*.

[CIT0026] Løgstrup K. E (1991). Den etiske fordring *[The ethical demand]*.

[CIT0027] Lomborg K, Bjørn A, Dahl R, Kirkevold M (2005). Body care experienced by people hospitalized with severe respiratory disease. Journal of Advanced Nursing.

[CIT0028] Martinsen B, Dreyer P (2012). Dependence on care experienced by people living with Duchenne muscular dystrophy and spinal cord injury. Journal of Neuroscience Nursing.

[CIT0029] Martinsen K (2012). Løgstrup & sygeplejen *[Loegstrup and nursing]*.

[CIT0030] McKinley S, Nagy S, Stein-Parbury J, Bramwell M, Hudson J (2002). Vulnerability and security in seriously ill patients in intensive care. Intensive Critical Care Nursing.

[CIT0031] McKinney A.A, Deeny P (2002). Leaving the intensive care unit: A phenomenological study of the patients’ experience. Intensive and Critical Care Nursing.

[CIT0032] Monrad M (2010). ldres oplevelse af at modtage hjælp [Elderly peoples experiences of receiving help]. Dansk sociologi.

[CIT0033] Polit D. F, Beck C. T (2010). Essentials of nursing research: Appraising evidence for nursing practice.

[CIT0034] Samuelson K. A. M (2011). Unpleasant and pleasant memories of intensive care in adult mechanically ventilated patients—Findings from 250 interviews. Intensive Critical Care Nursing.

[CIT0035] Schou L, Egerod I (2008). A qualitative study into the lived experience of post-CABG patients during mechanical ventilator weaning. Intensive Critical Care Nursing.

[CIT0036] Strandberg G, Jansson L (2003). Meaning of dependency on care as narrated by nurses. Scandinavian Journal of Caring Sciences.

[CIT0037] Strandberg G, Norberg A, Jansson L (2003). Meaning of dependency on care as narrated by 10 patients. Research and Theory for Nursing Practice.

[CIT0038] Sykepleiernes Samarbeid i Norden (SNN) (2003). Etiske retningslinier for sygeplejeforskning i Norden *[Ethical guidelines for nursing research in the Nordic countries]*.

[CIT0039] Tanggaard L, Brinkmann S, Tanggaard L, Brinkmann S (2010). Interviewet: Samtalen som forskningsmetode *[The interview: The dialogue as research method]*. Kvalitative metoder: en grundbog *[Qualitative methods: A basic book]*.

[CIT0040] Wardle B (1997). Dependency scoring in a critical care area: A direct nursing assessment method. Intensive and Critical Care Nursing.

